# Determining the contributions of protein synthesis and breakdown to muscle atrophy requires non‐steady‐state equations

**DOI:** 10.1002/jcsm.12772

**Published:** 2021-08-21

**Authors:** Kamil A. Kobak, Marcus M. Lawrence, Gavin Pharaoh, Agnieszka K. Borowik, Frederick F. Peelor, Patrick D. Shipman, Timothy M. Griffin, Holly Van Remmen, Benjamin F. Miller

**Affiliations:** ^1^ Aging and Metabolism Research Program Oklahoma Medical Research Foundation Oklahoma City OK USA; ^2^ Laboratory for Applied Research on Cardiovascular System, Department of Heart Diseases Wroclaw Medical University Wroclaw Poland; ^3^ Department of Kinesiology and Outdoor Recreation Southern Utah University Cedar City UT USA; ^4^ Department of Mathematics Colorado State University Fort Collins CO USA; ^5^ Oklahoma City VA Medical Center Oklahoma City OK USA

**Keywords:** Muscle atrophy, Denervation, Isotope labelling, Deuterium oxide, Protein synthesis, Protein degradation

## Abstract

**Background:**

Ageing and cachexia cause a loss of muscle mass over time, indicating that protein breakdown exceeds protein synthesis. Deuterium oxide (D_2_O) is used for studies of protein turnover because of the advantages of long‐term labelling, but these methods introduce considerations that have been largely overlooked when studying conditions of protein gain or loss. The purpose of this study was to demonstrate the importance of accounting for a change in protein mass, a non‐steady state, during D_2_O labelling studies while also exploring the contribution of protein synthesis and breakdown to denervation‐induced muscle atrophy.

**Methods:**

Adult (6 months) male C57BL/6 mice (*n* = 14) were labelled with D_2_O for a total of 7 days following unilateral sciatic nerve transection to induce denervation of hindlimb muscles. The contralateral sham limb and nonsurgical mice (*n* = 5) were used as two different controls to account for potential crossover effects of denervation. We calculated gastrocnemius myofibrillar and collagen protein synthesis and breakdown assuming steady‐state or using non‐steady‐state modelling. We measured RNA synthesis rates to further understand ribosomal turnover during atrophy.

**Results:**

Gastrocnemius mass was less in denervated muscle (137 ± 9 mg) compared with sham (174 ± 15 mg; *P* < 0.0001) or nonsurgical control (162 ± 5 mg; *P* < 0.0001). With steady‐state calculations, fractional synthesis and breakdown rates (FSR and FBR) were lower in the denervated muscle (1.49 ± 0.06%/day) compared with sham (1.81 ± 0.09%/day; *P* < 0.0001) or nonsurgical control (2.27 ± 0.04%/day; *P* < 0.0001). When adjusting for change in protein mass, FSR was 4.21 ± 0.19%/day in denervated limb, whereas FBR was 4.09 ± 0.22%/day. When considering change in protein mass (k_syn_), myofibrillar synthesis was lower in denervated limb (2.44 ± 0.14 mg/day) compared with sham (3.43 ± 0.22 mg/day; *P* < 0.0001) and non‐surgical control (3.74 ± 0.12 mg/day; *P* < 0.0001), whereas rate of protein breakdown (k_deg,_ 1/t) was greater in denervated limb (0.050 ± 0.003) compared with sham (0.019 ± 0.001; *P* < 0.0001) and nonsurgical control (0.023 ± 0.000; *P* < 0.0001). Muscle collagen breakdown was completely inhibited during denervation. There was a strong correlation (*r* = 0.83, *P* < 0.001) between RNA and myofibrillar protein synthesis in sham but not denervated muscle.

**Conclusions:**

We show conflicting results between steady‐ and non‐steady‐state calculations on myofibrillar protein synthesis and breakdown during periods of muscle loss. We also found that collagen accumulation was largely from a decrease in collagen breakdown. Comparison between sham and non‐surgical control demonstrated a crossover effect of denervation on myofibrillar protein synthesis and ribosomal biogenesis, which impacts study design for unilateral atrophy studies. These considerations are important because not accounting for them can mislead therapeutic attempts to maintain muscle mass.

## Introduction

Protein mass is maintained through the balance between protein synthesis and degradation. Because of technical challenges associated with measurement of protein breakdown, most of what we understand about protein turnover has come from measurements of protein synthesis. There is increasing use of deuterium oxide (D_2_O) labelling to measure protein synthesis because of several advantages it has over labelled amino acids.^1^ An advantage of D_2_O is that it can be provided orally to maintain a steady‐state body water enrichment, which facilitates long‐term labelling over days and weeks. This long‐term labelling helps to capture slowly synthesizing proteins such as myofibrillar and extracellular matrix (ECM) proteins. However, labelling over extended time periods (e.g. days to weeks) creates methodological challenges that are usually negligible when performing short‐term labelling.

An assumption for isotope studies is that the size of the protein pool of interest is constant over the experimental period.^2^ However, when performing measurements over days to weeks, there are many conditions, such as disuse atrophy, where the protein product pool is not constant over time. Most published studies using D_2_O do not account for this lack of steady state. In such cases, not accounting for a changing product pool size can substantially alter synthesis rate calculations. Because of this concern, we developed a model^3^, ^4^ that is similar to others^5^, ^6^ to account for changes in protein product pool size during non‐steady conditions. An additional benefit of this strategy is that rates of protein breakdown can be calculated to understand individual contributions of changes in synthesis and degradation during gain or loss of mass.

Non‐steady‐state model calculations are critical for various conditions such as ageing, disuse and systemic diseases that lead to loss of muscle mass. Loss of muscle mass and function significantly reduces quality of life and contributes to increased morbidity and all‐cause mortality in older people with or without chronic diseases.^7^ Among numerous mechanisms implicated in age‐related muscle atrophy, loss of innervation is one of the most important factors responsible for muscle wasting during disuse, ageing or neurodegenerative diseases.^8^ One model used for disuse atrophy and muscle loss is denervation by sciatic nerve transection. This model induces an expeditious loss of muscle mass and has been used to study mechanisms of innervation on muscle atrophy and mass changes.^9^, ^10^

Short‐term flooding dose radioisotope studies by Goldspink showed both increased and decreased synthesis and degradation rates in atrophied muscle that changed at different timepoints after denervation surgery.^11^, ^12^ However, short‐term (minutes to hours) labelling can bias tissue results to proteins that are most abundant or have the fastest synthesis rates.^4^ In addition, they capture a rather short snapshot in time where synthesis rates are influenced by physiological conditions at that exact time. As discussed, D_2_O facilitates measurement of cumulative responses over an extended period. In a recent investigation, Langer et al. conducted D_2_O labelling from 14 to 28 days after nerve damage to study effects of denervation‐induced muscle loss on myofibrillar protein synthesis.^13^ Four weeks after surgery, tibialis anterior (TA) muscle mass decreased 66% in the denervated limb vs. sham control. The authors used a steady‐state model to conclude that myofibrillar protein synthesis was increased in the denervated TA muscle; however, the magnitude of change in protein synthesis calculations directly depends on protein pool size. If, as expected, some loss of protein mass occurred during the labelling period at 14–28 days after nerve damage, the myofibrillar protein synthesis rates are likely different from what were reported.

Like others, the study of Langer et al.^13^ used the contralateral limb, which remained innervated, as the control muscle. However, there is a growing evidence for a so‐called crossover effect in contralateral limbs in models of muscle loss and gain. For example, Liu and Thomson^14^ reported a significant increase of proteasome activities in contralateral‐innervated muscles after 7 and 14 days of nerve transection as compared with muscles from non‐surgical mice. In addition, studies using a massage mimetic after disuse atrophy show an increased muscle regrowth in the non‐massaged contralateral limb.^3^ Therefore, in the current study, we tested for differences between the contralateral sham‐operated limb and a non‐surgical control limb.

The purposes of this study were to (1) determine contributions of myofibrillar and collagen protein synthesis and protein breakdown to denervation‐induced muscle atrophy using mathematical models that account for changes in the protein pool size over time and (2) investigate the ‘crossover effect’ by including a comparison of contralateral‐innervated muscles to muscles from a non‐surgical control. To address these goals, we used long‐term D_2_O labelling during a period of atrophy from sciatic nerve transection. In addition to protein turnover, we included measurements of ribosomal biogenesis to add insight to the pathophysiology of denervation atrophy. We hypothesized that synthesis and degradation rates would quantitatively and qualitatively change depending if change in protein mass is accounted for and that there would be a crossover effect of denervation to sham control muscles.

## Methods

### Animals

All experiments were conducted following a protocol approved by the Oklahoma Medical Research Foundation's (OMRF) Institutional Animal Care and Use Committee prior to any animal work. Six‐month‐old male C57BL/6J mice (Jackson Laboratories) were group housed in the AAALAC‐accredited OMRF vivarium on a 14:10 h light/dark cycle with *ad libitum* access to water and food (*n* = 14 for denervation and *n* = 5 for non‐surgical control).

### Experimental model

Sciatic nerve transection was performed on experimental mice as previously described.^9^ Animals were anaesthetized using constant‐flow isoflurane inhalation anaesthesia. In each hindlimb (at the femur), a small incision was made, and sciatic nerves were isolated. In left legs, sciatic nerves were severed, and a 5‐mm section of the sciatic nerve was removed. Nerve ends were folded back and closed with reabsorbable sutures to prevent nerve regrowth. Sham surgery was performed on contralateral right limbs, which served as the intra‐animal control. Additionally, non‐surgical age and weight‐matched controls were added to investigate potential crossover effects. After 7 days of D_2_O labelling (described below), gastrocnemius muscles from both limbs were quickly removed, trimmed of excess fat and connective tissues, weighed and flash‐frozen in liquid nitrogen.

### Deuterium oxide labelling protocol

**Figure 1:**
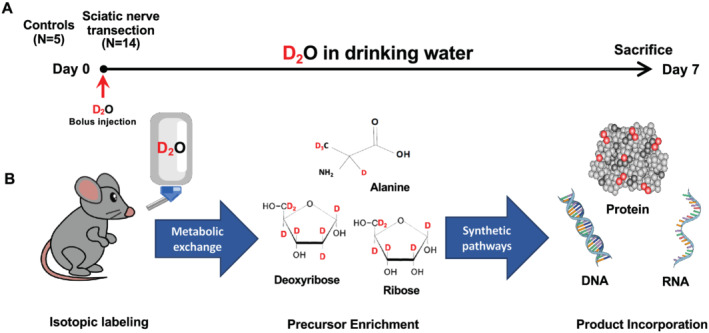
Schematic representation of the study protocol (*A*). Schematic principles of D_2_O labelling (*B*).

Mice were labelled as previously described.^3^, ^15^ Briefly, to initiate labelling during denervation surgery, mice were administered a bolus dose of isotonic deuterium oxide (Sigma‐Aldrich, D_2_O, 99%) equivalent to 5% of the body water pool. For the remainder of the 7‐day labelling period, mice were allowed free access to drinking water enriched 8% with D_2_O. At the end of 7 days, mice were euthanized, and tissue was collected (*Figure* 1A). Principles of D_2_O labelling are presented on *Figure* 1B.

### Protein turnover determination

For analysis of protein turnover, tissues were fractionated according to our previously published procedures, including collagen and RNA.^3^, ^16^ After homogenization, subcellular fractions were isolated via differential centrifugation as previously described.^3^, ^16^ Proteins were hydrolysed, and the pentafluorobenzyl‐*N*,*N*‐di (pentafluorobenzyl) derivative of alanine was analysed on an Agilent 7890A GC (Agilent, Santa Clara, USA) coupled to an Agilent 5975C MS (Agilent, Santa Clara, USA).^3^, ^16^

### Ribosomal biogenesis and cell proliferation determination

For analysis of ribosomal biogenesis, total RNA isolation was performed according to our previously published procedures^15^, ^16^ using 10–15 mg of skeletal muscle and TRIzol (Thermo Fisher, Rockford, IL, USA). The upper aqueous layer was isolated for RNA, whereas the bottom TRIzol phase was used to isolate proteins for the hydroxyproline assay. Total DNA was isolated using AllPrep DNA/RNA Micro Kit (Qiagen, Germany) on 5 mg of skeletal muscle. RNA and DNA concentrations were determined using a NanoDrop (Thermo Fisher Scientific). Isolated RNA and DNA were hydrolysed, derivatized and analysed on an Agilent 7890A GC coupled to an Agilent 5975C MS according to our previously published methods,^3^, ^16^ with subsequent analysis using ChemStation software. All analyses were corrected for abundance with an unenriched pentafluorobenzyl triacetyl purine ribose/deoxyribose derivative standard.

### Body water determination

To determine body water enrichment, 120 μL of plasma was placed in the inner well of an O‐ring cap of inverted screw‐capped tubes and placed in a heat block for overnight distillation at 80°C. Distilled samples were diluted 1:300 in ddH_2_O and analysed on a liquid water isotope analyser (Los Gatos Research, Los Gatos, CA, USA) against a standard curve prepared with samples containing different concentrations of D_2_O.^16^

### Collagen content

After TRIzol extraction, we precipitated the protein fraction and centrifuged. Protein pellets were washed three times with 0.3 M guanidine‐HCl in 95% ethanol and once in 100% ethanol. Pellets were dried and then solubilized in NaOH with heat. Protein was hydrolysed by incubation for 24 h at 110°C in 6 N HCl. Chloramine‐T and Ehrlich's reagent were added to form a chromophore that was measured at 558 nm.^17^ Collagen content was also measured as a fraction of total muscle area using picrosirius red staining, as described previously.^18^ These analyses were performed on gastrocnemius muscle cross‐sections of 10 μm that were cut midbelly using a cryostat at −20°C (Thermo Fisher). Whole stained cross sections were imaged at 10× magnification using bright field and light on a confocal microscope (Zeiss LSM 710) using the tiling function (Zeiss Zen Blue software, OMRF Imaging Core). To evaluate collagen content, ImageJ software was used to determine the percent area of red staining (collagen) relative to whole muscle cross section of bright‐field images.

### Calculations

For steady‐state calculations, the newly synthesized fraction (*f*) of proteins was calculated from enrichment of alanine bound in muscle proteins over the entire labelling period, divided by the true precursor enrichment (*p*), using plasma D_2_O enrichment with mass isotopomer distribution analysis (MIDA) adjustment. For non‐steady‐state calculations, we used calculations derived in our previously published papers.^3^, ^4^ The mass of protein at time *t*, *P(t)*, obeys the differential equation:

$$\frac{dP}{dt}=k_{\mathrm{syn}}-k_{\deg}P\left(t\right)$$

where *k*_*syn*_ is synthesis rate, with dimensions of mass over time, and *k*_*deg*_ is degradation constant, with dimensions of inverse time. Because *dP/dt* has dimensions of mass/time, *k*_*syn*_ and *k*_*deg*_ P must also have dimensions of mass/time. Because *P* has dimensions of mass, this means that *k*_*deg*_ must have dimensions of inverse time. The solution to this differential equation gives an expression for total mass as:

$$P\left(t\right)=P_{0}e^{-k_{\deg}t}+P_{\mathrm{eq}}\left(1-e^{-k_{\deg}t}\right).$$

In this expression, *P*_0_ = *P*(0) is initial mass and *P*_*eq*_ is equilibrium mass. We used the sham gastrocnemius mass and hydroxyproline concentration to calculate the change in myofibrillar and collagen protein mass, respectively. From equations derived in Miller et al.,^3^, ^4^ we have the following expressions: *P*_*eq*_ is equal to the ratio of *k*_*syn*_ to *k*_*deg*_, so that

$$k_{\mathrm{syn}}=k_{\deg}P_{\mathrm{eq}}$$

The degradation constant in terms of precursor enrichment *E* ^*^ and enrichment *E*(*t*) of the mass at time *t* is

$$k_{\deg}=-\frac{1}{t}\ln\left[\left(1-\frac{E\left(t\right)}{E^{*}}\right)\frac{P\left(t\right)}{P_{0}}\right]$$

This fractional breakdown rate is defined to be

$$\mathrm{FBR}=k_{\deg}\left(100\right)$$

The fractional synthesis rate is

$$\mathrm{FSR}=\frac{100}{t}\frac{1}{P_{0}}\left[P_{0}-P\left(t\right)\left(1-\frac{E\left(t\right)}{E^{*}}\right)\right]$$

Denervation did not result in significant change of total DNA or RNA content in gastrocnemius, and therefore, we used standard RNA FSR calculations as previously published^15^, ^16^ with MIDA adjustment of the equilibration of the enrichment of the body water pool with purine ribose.

### Statistical analyses

We used GraphPad Prism Version 8.3.0 (GraphPad Software, San Diego, California USA). The Kolmogorov–Smirnov test was used to verify the normality of the distribution of continuous variables. Two‐group comparisons were conducted using paired *t*‐test (two‐tailed). Outliers in collagen synthesis and degradation rates were removed from the analyses using ROUT method with Q = 1%. The cause of the outlier values were erroneous hydroxyproline results from performing the assay on a low amount of remaining tissue. Pearson's correlation coefficient (*r*) was calculated between the RNA fraction new and protein synthesis and degradation rates (*k*_*syn*_ and *k*_*deg*_). All values were reported as mean ± standard error of the mean (SEM), and statistical significance was set at *P* < 0.05.

## Results

### Muscle denervation changes protein synthesis and degradation

**Figure 2:**
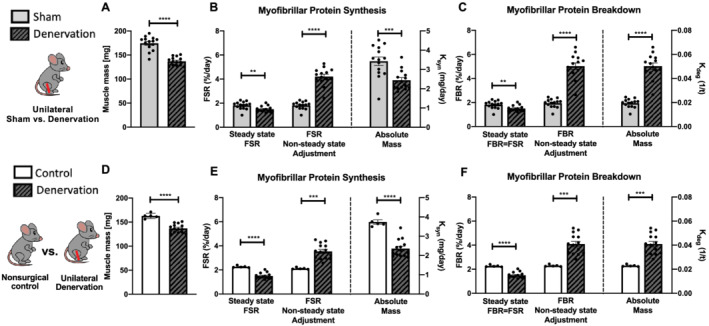
Effects of sciatic nerve dissection on myofibrillar protein turnover in gastrocnemius muscle as compared with two types of control: sham leg in unilateral model of denervation (*A–C*) and non‐surgical control (*D,E*). Gastrocnemius muscle weight (*A,D*). Myofibrillar protein synthesis (*B,E*) and degradation (*C,F*) calculated with three different approaches: steady‐state FSR, non‐steady‐state adjustment and absolute mass. FSR, fractional synthesis rate; FBR, fractional breakdown rate; *k*_*syn*_, synthesis rate; *k*_*deg*_, degradation rate; MYO, myofibrillar fraction.

Because the sham leg showed differences from a non‐surgical control (*Figure* S1), we compared denervation limb data separately to both sham leg and non‐surgical control leg data. Seven days after sciatic nerve transection, gastrocnemius muscle mass was approximately 20% lower when compared with sham limb (*Figure* 2A) and 16% lower when compared with non‐surgical control (*Figure* 2D). When using steady‐state FSR calculations in denervated muscle, there was a 24% lower protein synthesis compared with sham and 34% less when compared with non‐surgical control (*Figure* 2B and 2E). Because steady‐state calculations assume that synthesis equals breakdown, the breakdown rates were different from sham and non‐surgical control by the same amount (*Figure* 2C and 2F). As muscle mass is determined mostly by myofibrillar proteins, we used the non‐steady‐state model to calculate changes in myofibrillar turnover. FSR based on a non‐steady‐state adjustment was twofold higher when compared with both sham and non‐surgical control (*Figure* 2B and 2E). FBR based on non‐steady‐state adjustment model was 2.6‐fold and 1.8‐fold higher protein breakdown as compared with sham and non‐surgical control, respectively (*Figure* 2C and 2F). Finally, calculation of *k*_*syn*_ in the denervated leg showed an almost ~25% lower myofibrillar protein synthesis rate (*Figure* 2B) and a 2.5‐fold greater myofibrillar protein degradation rate as compared with sham (*Figure* 2C). When compared with nonsurgical control, loss of muscle mass was caused by both 35% lower synthesis rate *k*_*syn*_ (*Figure* 2E) and almost twofold greater k_deg_ (*Figure* 2F) of myofibrillar proteins.

Collagen accumulation in denervated muscle is from inhibition of collagen degradation.

**Figure 3:**
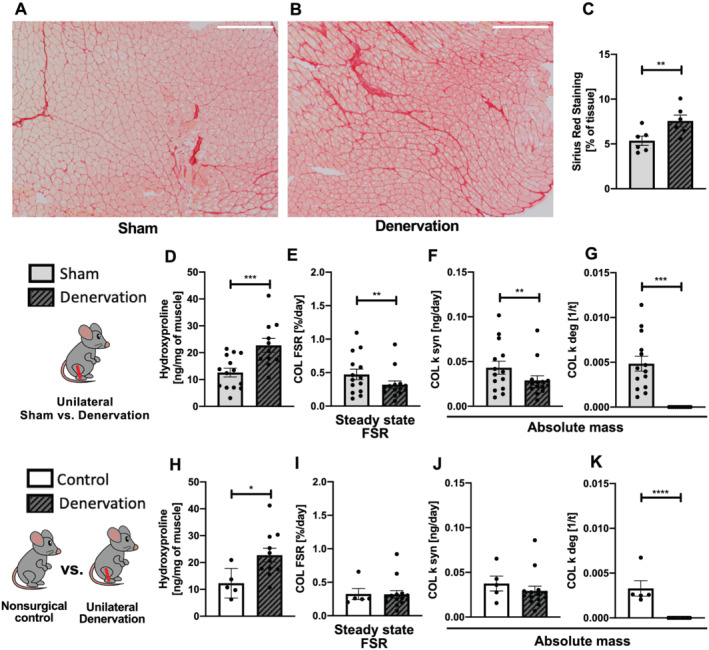
Effects of sciatic nerve dissection on collagen protein turnover in gastrocnemius muscle as compared with two types of control: sham leg in unilateral model of denervation (*A–G*) and non‐surgical control (*H–K*). Collagen accumulation in denervated muscle showed with picrosirius red staining (*A–C*). Collagen content in muscle measured by hydroxyproline assay (*D,H*). Collagen turnover calculated by steady‐state FSR (*E,I*) and non‐steady‐state (*F,G,J,K*) approaches. FSR, fractional synthesis rate; *k*_*syn*_, synthesis rate; *k*_*deg*_, Degradation rate; COL, collagen fraction.

Both picrosirius red staining (*Figure* 3A–C) and hydroxyproline assay results (*Figure* 3D) showed a significantly higher collagen content in denervated gastrocnemius muscle as compared with sham, indicating a non‐steady state in collagen protein pool. When calculated with standard steady‐state FSR calculations, collagen synthesis was slightly lower in denervated limb as compared with sham (*Figure* 3E). When non‐steady‐state model was applied to calculate *K*_*syn*_ and *K*_*deg*_, we found slightly decreased synthesis rate (*Figure* 3F) accompanied by completely inhibited degradation (*Figure* 3G) as compared with sham. When compared with non‐surgical control, the collagen content in denervated muscle was also greater (*Figure* 3H). Although no significant differences were observed in collagen FSR (*Figure* 3I) and synthesis rate (*Figure* 3J), collagen degradation was also completely inhibited in denervated gastrocnemius (*Figure* 3K).

### Changes in ribosomal biogenesis accompany denervation atrophy

**Figure 4:**
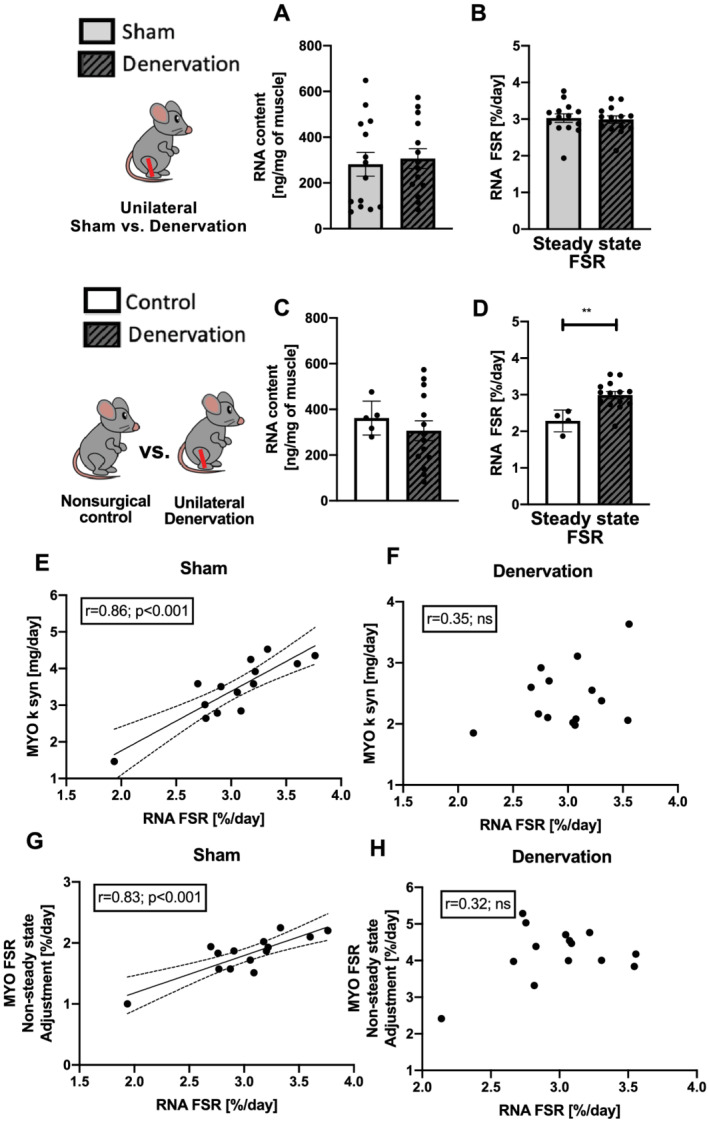
Effects of sciatic nerve dissection on ribosomal biogenesis in gastrocnemius muscle as compared with two types of control: sham leg in unilateral model of denervation (*A,B*) and non‐surgical control (*C,D*). RNA content (*A,C*) and synthesis (*B,D*) in gastrocnemius muscle. Correlation between RNA FSR and MYO synthesis rate and non‐steady‐state adjustment FSR in sham (*E,G*) and denervated (*F,H*) muscle. FSR, fractional synthesis rate; *k*_*syn*_, synthesis rate, MYO, myofibrillar fraction.

There were no significant differences in RNA content (*Figure* 4A) or RNA synthesis rate (*Figure* 4B) between sham and denervated gastrocnemius, suggesting no change in ribosomal biogenesis. There was a strong correlation between RNA synthesis rate and myofibrillar protein synthesis in muscle from the sham limb (*Figure* 4E and 4G) but not in denervated muscle (*Figure* 4F and 4H). When compared with non‐surgical control muscle, there was no difference in RNA content (*Figure* 4C), but ribosomal biogenesis was greater in denervated muscle (*Figure* 4D). This difference in RNA synthesis comparing denervated muscle with sham vs. non‐surgical control muscle is due to a greater rate of RNA synthesis of the sham vs. non‐surgical control muscle. Thus, these data suggest that sham muscle might undergo compensatory changes and not be a true control for denervated muscle obtained from the contralateral limb.

No significant differences in DNA content were observed between denervated and sham gastrocnemius (*Figure* S2A), indicating steady state in the DNA pool. However, there was a fourfold higher DNA synthesis in denervated muscle (*Figure* S2B). Surprisingly as compared with non‐surgical control, DNA content was significantly higher in denervated muscle (*Figure* S2B), which justified the non‐steady‐state approach. The increase of DNA turnover was caused by both greater synthesis rate (*Figure* S2E) and lower degradation (*Figure* S2F). Steady‐state FSR model calculations also showed increased DNA synthesis (*Figure* S2C).

## Discussion

The primary goal of this study was to demonstrate the importance of accounting for changes in protein pool size when performing long‐term isotope labelling during conditions when total protein mass changes. We used denervation‐induced muscle atrophy because of its reproducible loss of muscle protein mass and to compare our outcomes with others in the literature. Using D_2_O labelling and non‐steady‐state modelling, we showed that compared with control, there was greater myofibrillar protein breakdown with muscle denervation, but the interpretation of the change in protein synthesis depended on whether protein was considered as a fraction (FSR) or as an absolute quantity (*K*_*syn*_). Furthermore, in denervated muscle, collagen degradation was absent (or not measurable) without a change in collagen synthesis, which resulted in greater collagen content. Overlooking the non‐steady‐state condition would have led to the conclusion that collagen synthesis was higher in the denervated limb compared with control. Finally, we showed that there were differences in myofibrillar protein synthesis and ribosomal biogenesis when the sham operated leg was used compared with a separate non‐operated control, which argues for the use of an appropriate control in studies of muscle atrophy.

### The importance of steady or non‐steady‐state considerations

A fundamental assumption of isotope studies is that the size of the protein pool does not change during the labelling period.^2^ This assumption is not true when the size of the muscle changes, for example, atrophy or hypertrophy, during the period of labelling. Although a change in protein mass is not a concern with short‐term (e.g. minutes to hours) labelling studies, it is an important consideration in studies using long‐term labelling (e.g. days to weeks) afforded by D_2_O labelling. Unfortunately, to date, this important consideration has largely been overlooked.

**Figure 5:**
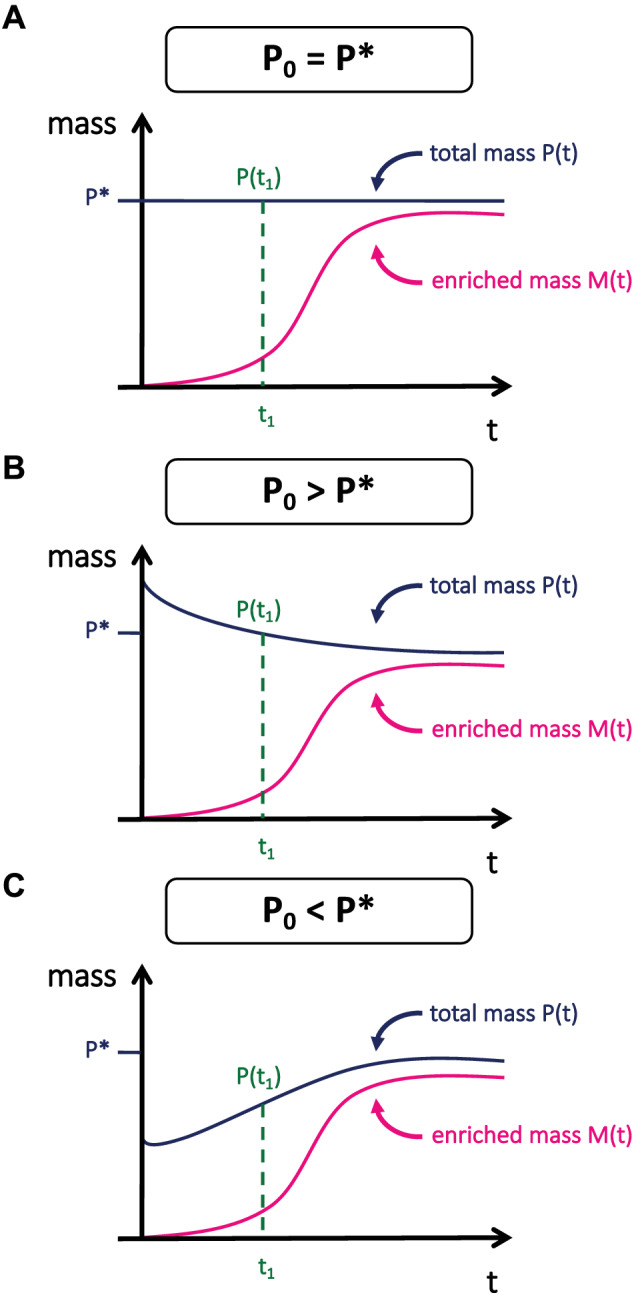
Implications of not accounting for change of mass on calculated protein FSR. If it is assumed that the mass of enriched protein is zero, M(0) = 0, *K*_*syn*_ and *K*_*deg*_ determine the change in the mass of enriched protein over time. Further, the total (enriched and unenriched) protein mass, *P*(*t*), is determined by *K*_*syn*_ and *K*_*deg*_ along with the initial protein mass *P*(0) and eventually reaches an equilibrium mass (P*). Importantly, the total enriched protein mass at a given time, *M*(*t*), is independent of the initial total protein mass, *P*(0). Therefore, to calculate the fraction new that is independent of *P*(0), the difference in protein mass, *P*(t), from P*, must be accounted for. (A) Protein mass is in a steady state. (B) Protein mass is decreasing, as during denervation. (C) Protein mass is increasing as during hypertrophy.

Skeletal muscle atrophy is a physiological non‐steady state caused by an imbalance in myofibrillar protein turnover, namely, when protein degradation is higher than synthesis. In the present study, the protein pool size was assumed to be proportional to muscle mass, which changed significantly (~20% lower in denervated muscle) during the 7‐day labelling period. This change in mass is in agreement with other studies using this model.^9^ Not accounting for this change in protein pool size has a mathematical consequence when calculating FSR, which we have previously discussed,^4^ and is illustrated in *Figure* 5. To explain, FSR is calculated assuming that the initial total mass remains constant and that synthesis is a zero‐order function where the mass of enriched protein over time does not depend on the initial mass. However, during a period of atrophy, the eventual equilibrium mass is less than the initial mass; thus, the calculation of FSR (the fraction of total protein mass that is new) will therefore be smaller than if one corrected for change in mass. The result is that if the non‐steady state is not accounted for, a calculation of FSR during muscle loss or muscle gain will be under‐ or overestimated, respectively.

Traditional measurements of FSR are reported as %/hr or %/day as there is a fraction of the protein pool that is new over a period of time. However, during a period where there is a change in protein mass, the fraction of protein new is not necessarily reflective of differences in the absolute amount of protein being made. As an example, 10% new of 100 proteins (i.e. 10 proteins) is far less protein in an absolute sense than 10% new of 500 proteins (i.e. 50 proteins). For some comparisons, the fraction of the protein pool that is turning over is the important factor. However, when protein mass is changing, this fraction is less informative. As illustrated in *Figure* 2, the FSR calculated with non‐steady‐state equations is greater in the denervated leg compared with control, but this fraction is of a lower total protein mass. When we calculated *k*_*syn*_, which provides units of mg/day, the total amount of protein synthesized in the denervated muscle is actually less than the control. The same is not true for breakdown rates where FSR and *k*_*deg*_ both indicate greater rates in the denervated leg compared with control. Therefore, the conclusion of whether denervation increases or decreases protein synthesis depends if one considers the fraction of the pool or the total amount of protein.

The previously mentioned study of Langer et al.^13^ showed an increase of myofibrillar protein synthesis in denervated TA muscle. The methods presented in the study calculated FSR assuming steady‐state conditions, which implies that protein synthesis equals breakdown. However, one of the main conclusions of the paper was that protein breakdown must have been higher than protein synthesis to cause the muscle loss. Therefore, it was recognized that there was not a steady state, but the calculations did not account for it. The isotopic labelling in the aforementioned study started 14 days after denervation and concluded 14 days later. Although it is possible that the muscle mass was in a steady state during the final 14 days, other studies have indicated that there is still a significant (~40%) loss of myofibrillar protein during this period of time.^19^ It is therefore likely that FSR reported in the study is not indicative of the actual response to unloading. Further, as we demonstrate, the absolute quantity of protein synthesized was likely less, but could not be determined when steady state is assumed.

There are practical consequences of not understanding the true impact of protein synthesis vs. protein breakdown when designing therapeutic approaches. Currently, there are several potential targets for the treatment of muscle loss that include stimulating protein synthesis (e.g. mammalian target of rapamycin activators or growth factors) or inhibit protein degradation (e.g. myostatin/activin A antagonists).^20^ In the present study, we showed that both lowered protein synthesis and higher protein breakdown are contributors to the loss of muscle mass in a denervation model of atrophy. These findings are in contrast to the study of Langer et al.^13^ that concluded that the loss must be due solely to a large increase in protein breakdown. Therefore, the recommended therapeutic approach would differ whether the non‐steady state is accounted for or not.

### Collagen turnover in denervation atrophy

Long‐term D_2_O labelling studies are well suited for the measurement of slowly synthesizing proteins, such as collagen—the main component of muscle ECM proteins. ECM plays a crucial role in muscle fibre force transmission, maintenance and repair.^21^ Besides the loss of muscle mass, collagen accumulation and remodelling have been implicated as pathological features responsible for loss of muscle function in different atrophying conditions.^22^, ^23^ In particular, disuse and muscle ageing are associated with fibrotic collagen ECM deposition that is often unresolvable and may further stiffen muscle ECM or even promote satellite cell fibrogenic conversion.^23^, ^24^ Therefore, maintained collagen turnover is essential to maintain proper muscle function. Studies measuring collagen mRNA levels in young and old mice suggest that the age‐related accumulation of collagen is not a result of increased synthesis; rather, it is more likely due to impaired degradation caused by increased crosslinking of the collagen molecules with age.^25^ However, assessing the collagen turnover by measuring mRNA levels may be limited because of comprehensive post‐transcriptional mechanisms involved in collagen synthesis.^26^, ^27^ Although short‐term amino‐acid labelling studies showed some adaptive changes in collagen synthesis,^28^, ^29^ these labelling approaches biased results to rapidly turning over proteins, which underestimate important changes that accumulate over time.^4^

The present study showed a significant deposition of collagen at 7 days after sciatic nerve dissection, which agrees with studies that showed accumulation at 2 weeks after denervation.^30^, ^31^ Using our D_2_O labelling, we were able to confirm that the accumulation of collagen in this model of disuse atrophy is not caused by increased collagen synthesis, but rather a complete inhibition of collagen breakdown. In the context of evidence that restoring muscle mass might not be enough to maintain muscle function,^32^ our findings indicate that inhibiting collagen degradation may be important target to maintain muscle quality after disuse.

### Ribosomal biogenesis in denervation

The decline in translational efficiency and capacity caused by a period of muscle disuse may be a therapeutic target to regain muscle in the period after disuse.^33^ Changes in ribosomal synthesis (ribosomal biogenesis) have been shown to correlate with changes in muscle protein synthesis during loading.^15^, ^16^, ^34^ In recent studies, we have shown that atrophy caused by disuse is associated with decreases in myofibrillar protein synthesis, increases in myofibrillar protein degradation and dramatically upregulated ribosomal degradation without changes to ribosome biogenesis.^16^, ^33^ In the current investigation, we observe that a correlation between ribosome biogenesis and myofibrillar protein synthesis exists in the sham limb (during normal loading conditions), but not in the denervated limb. Interestingly, this finding of correlated ribosomal biogenesis and protein synthesis during loading, but not unloading confirmed our previous finding in rats when looking at a period of hindlimb unloading vs. reloading. These repeated results lead us to believe that ribosomal biogenesis is an important component of normal loading and the gain of protein mass, but this relationship falls apart during disuse because of increases in ribosomal breakdown.

### The importance of appropriate control in unilateral models

Unilateral models of denervation are widely used for studies investigating the pathophysiology of muscle atrophy. However, there is a growing evidence for crossover effects of exercise, electrical stimulation, inflammation and injury in contralateral muscle.^14^, ^35^, ^36^ Although many previous studies have shown denervation‐induced alterations in proteostatic properties, most of them were using contralateral‐innervated muscle of a mouse with a nerve transection as experimental control.^37^, ^38^ Recently, Liu and Thomson^14^ showed that proteasomal activity and content are significantly increased in contralateral‐innervated muscles after 7 and 14 days of nerve transection as compared with muscles from non‐surgical mice. Moreover, when the non‐surgical mice were used as the experimental control, the robust increase in proteasome properties that were found in denervated muscles were not observed when the contralateral‐innervated muscle was used as a control. In the context of protein turnover, we recently extended these findings of a crossover effect when we showed increased myofibrillar protein synthesis in contralateral muscle of animals that were exposed to massage mimetic after disuse atrophy.^3^ Therefore, the use of an appropriate control is important for understanding the impact of disuse.

In the present study, we performed a direct comparison between the sham‐denervated limb and a non‐surgical control (*Figure* S1) and found some differences. Because of these differences, we calculated our outcomes using both controls as comparators to the denervated limb. When comparisons were made between the two types of controls and the denervated limb, there were different effects of denervation noted for collagen protein synthesis and ribosomal biogenesis. The current and previous data^14^ suggest that the crossover effect on contralateral muscles should be considered when the control is selected for unilateral model atrophy study. Using non‐surgical control allows to determine both effect of intervention and crossover effect on the contralateral limb.

### Study limitations and conclusions

This study has some limitations. First, the labelling period of 7 days after denervation surgery shows only the early effects of denervation, although this is a period where we expect rapid changes. Second, changes in myofibrillar protein content were determined by changes in muscle weight rather than a direct measure of total protein content. There are two justifications for this approach. First, it is estimated that 70% of muscle protein is myofibrillar, and changes in myofibrillar proteins are largely responsible for changes in mass.^39^ Second, a determination of protein concentration would need to be multiplied by muscle mass to get total protein mass, so any change in protein content would largely be driven by change in total mass. Third, the calculation of change in protein mass can be performed using mass per mass of tissue, or total mass. It is our contention that either approach can be appropriate based on the question of interest. Finally, our calculations rely on the change in protein mass. Therefore, the robustness of the calculation is limited by the ability to measure the changes in protein mass. During periods of very slow gain or loss, such that occurs over months to years, these changes would not be detectable, and steady state could be assumed. However, technologies such as quantitative proteomics should increase resolution to the level of individual proteins so more subtle changes can be assessed.

Collectively, our data demonstrate that when using long‐term labelling approaches, like those afforded by D_2_O, to measure protein turnover during a period when protein mass changes, it is critical to account for the changes in protein mass. Further, these assessments also require the appropriate controls. Using a denervation model of disuse, several protein fractions and a non‐surgical control, we demonstrated that not accounting for the changes in protein mass in tracer calculations can impact the qualitative and quantitative assessments of the causes of the changes in protein mass. Most importantly, these differences could mislead therapeutic attempts to preserve or increase muscle mass. Although we demonstrated the important implication of these changes during a period of muscle loss, the same principles apply to periods of muscle gain and to different protein pools.

## Conflict of interest

All authors declare that they have no conflict of interest.

## Supporting information


**Figure S1.** Comparison between sham leg in unilateral model of denervation and nonsurgical control. Gastrocnemius muscle mass (A) and myofibrillar protein fractional synthesis rate (B). Collagen (C), RNA (E) and DNA (G) content with corresponding fractional synthesis rates (D, F, H). MYO – myofibrillar fraction; COL – collagen fraction; FSR‐ fractional synthesis rate

**Figure S2.** Effects of sciatic nerve dissection on cell proliferation in gastrocnemius muscle as compared with two types of control: sham leg in unilateral model of denervation (A‐B) and nonsurgical control (C‐D). DNA content (A, C), synthesis (B, D‐E) and degradation (F) in gastrocnemius muscle.
