# Supplementary figures and images for: Determining the contributions of protein synthesis and breakdown to muscle atrophy requires non‐steady‐state equations
Source: J Cachexia Sarcopenia Muscle. 2021 Aug 21;12(6):1764–75. doi: 10.1002/jcsm.12772 (PMC8718081; doi:10.1002/jcsm.12772)

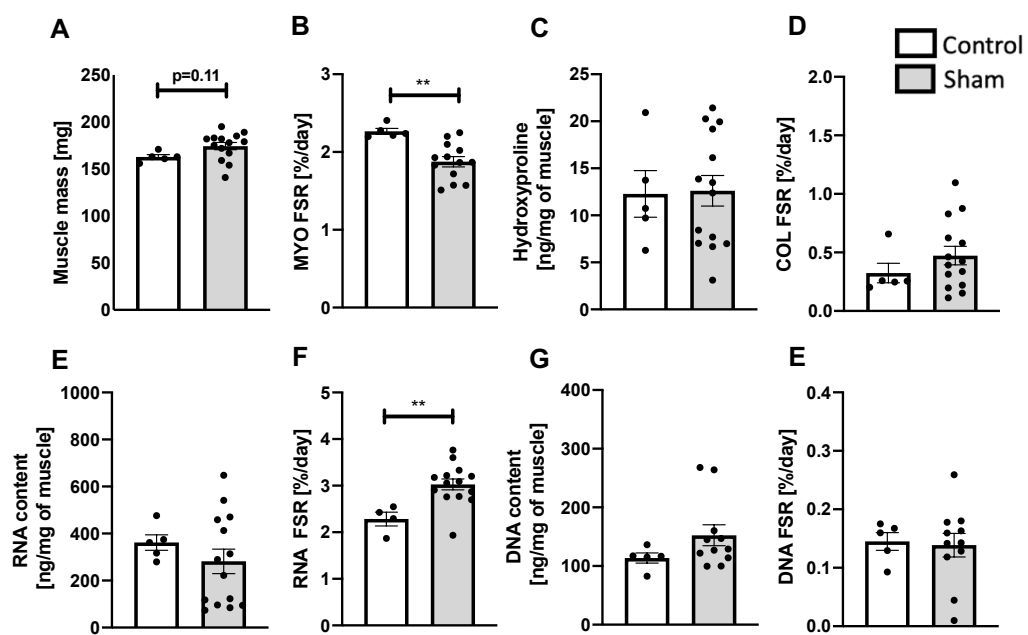

Supplement: Supplementary file 1 — Figure S1. Comparison between sham leg in unilateral model of denervation and nonsurgical control. Gastrocnemius muscle mass (A) and myofibrillar protein fractional synthesis rate (B). Collagen (C), RNA (E) and DNA (G) content with corresponding fractional synthesis rates (D, F, H). MYO – myofibrillar fraction; COL – collagen fraction; FSR‐ fractional synthesis rate [file JCSM-12-1764-s002.pdf]

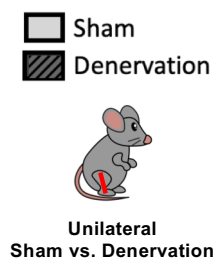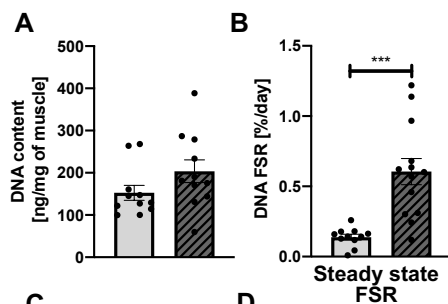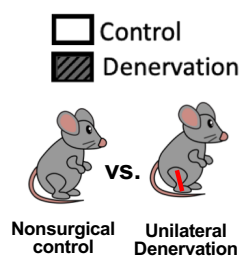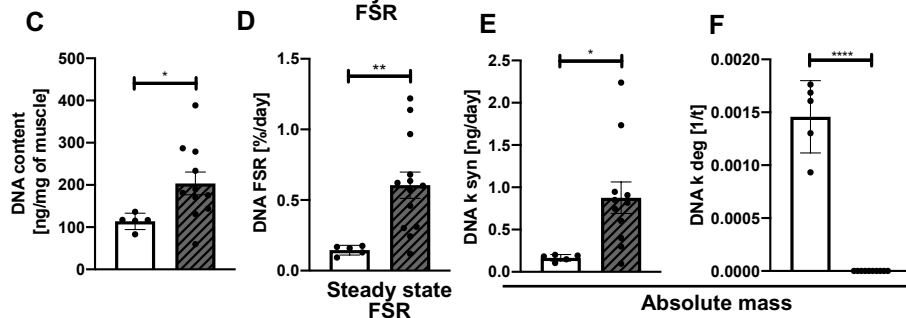

Supplement: Supplementary file 2 — Figure S2. Effects of sciatic nerve dissection on cell proliferation in gastrocnemius muscle as compared with two types of control: sham leg in unilateral model of denervation (A‐B) and nonsurgical control (C‐D). DNA content (A, C), synthesis (B, D‐E) and degradation (F) in gastrocnemius muscle. [file JCSM-12-1764-s001.pdf]
